# Tissue Distribution of Oleocanthal and Its Metabolites after Oral Ingestion in Rats

**DOI:** 10.3390/antiox10050688

**Published:** 2021-04-27

**Authors:** Anallely López-Yerena, Anna Vallverdú-Queralt, Olga Jáuregui, Xavier Garcia-Sala, Rosa M. Lamuela-Raventós, Elvira Escribano-Ferrer

**Affiliations:** 1 Department of Nutrition, Food Science and Gastronomy XaRTA, Faculty of Pharmacy and Food Sciences, Institute of Nutrition and Food Safety (INSA-UB), University of Barcelona, 08028 Barcelona, Spain; naye.yerena@gmail.com (A.L.-Y.); avallverdu@ub.edu (A.V.-Q.); lamuela@ub.edu (R.M.L.-R.); 2 CIBER Physiopathology of Obesity and Nutrition (CIBEROBN), Institute of Health Carlos III, 28029 Madrid, Spain; 3 Scientific and Technological Center of University of Barcelona (CCiTUB), 08028 Barcelona, Spain; ojauregui@ccit.ub.edu; 4 Biopharmaceutics and Pharmacokinetics Unit, Department of Pharmacy and Pharmaceutical Technology and Physical Chemistry, Institute of Nanoscience and Nanotechnology (IN2UB), Faculty of Pharmacy and Food Sciences, University of Barcelona, 08028 Barcelona, Spain; xgarciasala@ub.edu; 5 Pharmaceutical Nanotechnology Group I+D+I Associated Unit to CSIC, University of Barcelona, 08028 Barcelona, Spain

**Keywords:** extra virgin olive oil, polyphenols, metabolism, bioaccumulation, LC-ESI-LTQ-Orbitrap

## Abstract

Claims for the potential health benefits of oleocanthal (OLC), a dietary phenolic compound found in olive oil, are based mainly on in vitro studies. Little is known about the tissue availability of OLC, which is rapidly metabolized after ingestion. In this study, the distribution of OLC and its metabolites in rat plasma and tissues (stomach, intestine, liver, kidney, spleen, lungs, heart, brain, thyroid and skin) at 1, 2 and 4.5 h after the acute intake of a refined olive oil containing 0.3 mg/mL of OLC was examined by LC-ESI-LTQ-Orbitrap-MS. OLC was only detected in the stomach and intestine samples. Moreover, at 2 and 4.5 h, the concentration in the stomach decreased by 36% and 74%, respectively, and in the intestine by 16% and 33%, respectively. Ten OLC metabolites arising from phase I and phase II reactions were identified. The metabolites were widely distributed in rat tissues, and the most important metabolizing organs were the small intestine and liver. The two main circulating metabolites were the conjugates OLC + OH + CH_3_ and OLC + H_2_O + glucuronic acid, which may significantly contribute to the beneficial health effects associated with the regular consumption of extra virgin olive oil. However, more studies are necessary to determine the concentrations and molecular structures of OLC metabolites in human plasma and tissues when consumed with the presence of other phenolic compunds present in EVOO.

## 1. Introduction

According to the World Health Organization (WHO), the main causes of death worldwide in 2016 were ischemic heart disease, stroke, chronic obstructive pulmonary disease, lower respiratory infections, Alzheimer’s disease and other dementias and diabetes mellitus [1]. In 2018, the Mediterranean diet, a model of eating based on the traditional foods and drinks of the countries surrounding the Mediterranean Sea [2], was recommended by the WHO for its numerous health benefits, including effectiveness in reducing noncommunicable diseases [3]. The main source of fat in the Mediterranean diet is extra virgin olive oil (EVOO), whose minority compounds include phenolic substances such as phenolic acids, phenolic alcohols, flavonoids, secoiridoids and lignans [4,5]. Among the secoiridoids, which contain elenolic acid or its derivatives in their molecular structures [6], oleocanthal (OLC) has proven biological activity, including antioxidant and antimicrobial properties [7,8], anti-inflammatory effects (more potent than ibuprofen) [9], anticancer properties (melanoma, breast, liver and colon cancer and leukemia) [10,11,12] and neuroprotective effects [13,14].

Recognized by the human body as xenobiotics, phenolic compounds have a relatively low bioavailability compared with micro and macronutrients [15]. Bioavailability depends on multiple factors, including gastrointestinal digestion, absorption and metabolism, as well as tissue distribution [16], which plays a fundamental role in the pharmacokinetic behavior of drugs and other xenobiotics and conditions the pharmacological and toxicological responses of the organism [17]. Studies in animal models help to determine the tissue distribution and accumulation of compounds and their metabolites and identify potential sites of action [18]. To date, the extensive research on the bioavailability of phenolic compounds from EVOO has focused mainly on absorption or metabolism in humans [19,20,21,22,23,24] and rats [25,26,27,28]. Regarding OLC, a low intestinal absorption (16%) and intestinal metabolism in vivo, indicating limited systemic exposure, has been described by only one study [29], whereas none of the investigations aiming to understand the bioavailability of EVOO phenolic compounds have explored OLC tissue distribution. Recently, the OLC reactivity with amino acids with high preference to glycine was also reported [30].

It should also be noted that most of the in vitro studies on the health benefits of OLC do not take into account the matrix, the biological concentrations usually achieved when following a Mediterranean diet, metabolic transformations or the contemporary presence of more than one metabolite [31]. The current work is therefore an attempt to improve our understanding of the health effects of OLC through a tissue distribution study in rats after the oral administration of OLC-enriched refined olive oil (ROO). The distribution of OLC metabolites in multiple tissues and at different times post-administration was also evaluated.

## 2. Materials and Methods

### 2.1. Reagents and Materials

OLC (purity > 90) was purchased from PhytoLab GmbH (Vestenbergsgreuth, Germany), and its purity was verified by ^1^H NMR, as in a previous study [32]. The reagents of methanol, acetonitrile (ACN) and formic acid were purchased from Sigma-Aldrich. Ultrapure water was obtained using a Milli-Q purification system (Millipore, Bedford, MA, USA).

### 2.2. Animals and Diets

The studies were conducted following a protocol approved by the Animal Experimentation Ethics Committee of the University of Barcelona, Spain (trial no. CEEA 124/16) and Generalitat de Catalunya (no. 6435). Male Sprague Dawley rats (body weight 249 ± 8 g) were purchased from Envigo RMS Spain S.L. (Sant Feliu de Codines, Barcelona, Spain). The animals were acclimatized in animal housing in an air-conditioned room at 22 ± 2 °C under a 12 h/12 h cycle of light and darkness and given free access to a commercial diet (Teklad 2014, Envigo RMS Spain S.L.) and water for at least 10 days before the study. The rats were fasted for over 12 h before the experiments but were allowed free access to water.

### 2.3. Tissue Distribution Experiments

Before the distribution experiment, the ROO (vehicle) was analyzed by LC-ESI-LTQ-Orbitrap-MS to make sure it was free of phenolic compounds. The liquid–liquid extraction of phenolic compounds of ROO was performed following the previously described procedures [33]. The chromatographic and mass spectrometer conditions were the same as those used for the identification of metabolites (Section 2.3). After verifying that the ROO was free of phenols, OLC was added to obtain a final concentration of 0.3 mg/mL. The OLC concentration was chosen while taking into account both the OLC concentration in commercial EVOO (~300 mg/kg) and the daily ingestion of EVOO recommended by the European Food Safety Authority of at least 5 mg of hydroxytyrosol and its derivatives per 20 g of olive oil [34].

The rats were divided into four groups—a control group (no administration) and three dosed groups—to study the distribution of OLC at three different time points: 1, 2 and 4.5 h post-administration. On treatment day, the rats received the ROO containing OLC by oral gavage (2.5 mL/300 g body weight). The volume for gavage administration was established following a good practice guide for substance administration [35] and was lower than the maximum volume of lipid vehicles per kg of rat body weight that prevented elevated plasma corticosterone levels [36].

The rats were anesthetized with isoflurane (IsoFloR, Veterinaria Esteve, Barcelona, Spain) and euthanized by exsanguination via cardiac puncture at 1, 2 or 4.5 h after ROO ingestion. The control group was maintained under fasting conditions without olive oil ingestion and then similarly euthanized. The blood was collected in EDTA tubes, and plasma samples were obtained by centrifugation (3000× *g* for 10 min at 4 °C) and stored at −80 °C until analysis. Different tissues and organs (heart, liver, spleen, lungs, kidney, brain, thyroid, stomach, small intestine and skin) were removed from the rats and placed in pre-weighed tubes. The small intestine samples consisted of a pool of duodenum, jejunum and ileum segments, but the main region used was the jejunum. The procedure was to locate the plica duodenocolica [37] and, from there up to about 5 cm of the intestine before reaching the cecum, to remove it from the animal. The collected tissues were washed with physiological serum at 37 °C and placed between absorbent paper to remove the rest of the serum. In the case of the intestine, the luminal contents were first removed by applying pressure with the index finger and thumb, and then the lumen was washed by perfusing with the physiological serum at 37 °C. It was then placed between absorbent paper to remove the remains of the washings. In order to avoid compound oxidation, the collected tissues were kept on ice at all times. Finally, the samples were weighed and stored at −80 °C until analysis. For each group, four animals were used.

### 2.4. Liquid–Liquid Extraction for Bioanalysis

The tissues were homogenized with a small tissue disruptor (T 10 basic ULTRA-TURRAX^®^, IKA laboratory technology, Staufen Germany) in a 1:1 ratio (*w*/*v*) with water:ACN (1:1 (*v*/*v*) with 0.1% ascorbic acid). The samples were sonicated for 5 min in an ice bath and shaken for 1 min (Vortex-Genie 2). Each homogenate was centrifuged for 10 min (11,000× *g* at 4 °C). A volume of 100 µL of the upper layer was blended with cold ACN containing 2% formic acid in a 1:3 ratio (*v*/*v*) in order to precipitate the proteins [38]. The samples were homogenized for 1 min and kept at −20 °C for 20 min before centrifugation (11,000× *g* at 4 °C for 10 min). Finally, 100 µL of supernatant was transferred to vials for analysis. Three replicates were evaluated for each sample.

The plasma samples were thawed and centrifuged (11,000× *g* at 4 °C at 10 min). Plasma (100 µL) was mixed with cold ACN containing 2% formic acid in a ratio of 1:5 (*v*/*v*) to precipitate proteins, homogenized for 1 min and kept at −20 °C for 20 min. The samples were then centrifuged (11,000× *g* at 4 °C for 10 min), and finally, 100 µL of each organic phase was transferred to vials for analysis. Three replicates were evaluated for each sample.

### 2.5. LC-ESI-LTQ-Orbitrap-MS Equipment and Conditions

The LC system consisted of an Accela chromatograph (Thermo Scientific, Hemel Hempstead, UK) with a quaternary pump, a photodiode array detector (PDA) and a thermostated autosampler. The separations were carried out on an Acquity^TM^ UPLC^®^ BEH C_18_ pre-column (2.1 × 5 mm, i.d., 1.7 µm particle size) and an Acquity^TM^ UPLC^®^ BEH C_18_ column (2.1 × 100 mm, i.d., 1.7 µm particle size) (Waters Corporation^®^, Ireland). The mobile phases consisted of H_2_O (A) and methanol (B), both with 0.05% of formic acid. The elution was performed by means of an increasing linear gradient (*v*/*v*) of B (t (min), %B), as follows: (0, 0); (2, 0); (6, 53.6); (8, 100); (9, 100); (9.1, 0); and (11, 0). The injection volume was 5 µL, the flow rate was set to 0.6 mL/min, and the column temperature was 50 °C.

An LTQ Orbitrap Velos mass spectrometer (Thermo Scientific, Hemel Hempstead, UK) equipped with an ESI source in negative mode was used to obtain high-resolution mass spectra for the identification of OLC and its derivatives. The operation parameters were the following: source voltage = 4 kV and capillary temperature = 275 °C (FT automatic gain control (AGC) target 5·105 for MS mode and 5·104 for MSn mode). The sheath gas, auxiliary gas and sweep gas were set at 20, 10 and 2 arbitrary units, respectively. All samples were analyzed in Fourier transform mass spectrometry (FTMS) mode at a resolving power of 30,000 at 600 m/z, and data-dependent MS/MS events were acquired at a resolving power of 15.000 at 600 m/z. The most intense ions detected during FTMS-MS triggered data-dependent scanning. Ions that were not intense enough for a data-dependent scan were analyzed in MSn mode with the same orbitrap resolution (15,000 at 600 m/z). Precursors were fragmented by collision-induced C-trap dissociation with normalized collision energy (35 V) and an activation time of 10 ms. The mass range in FTMS mode was from 100 to 600 m/z. The system was controlled by XCalibur software v2.0.7 (ThermoFisher Scientific).

The OLC calibration curves were prepared in each isolated tissue matrix (heart, liver, spleen, lung, kidney, brain, thyroid, stomach, small intestine and skin) and in plasma (0.1–3 µg/mL). The concentrations of OLC metabolites were calculated using the peak area of the standard OLC and expressed as OLC equivalents. All calibration curves presented R2 > 0.98.

The elemental composition of each metabolite was selected according to the accurate masses and the isotopic pattern (through the Formula Finder feature in XCalibur software v2.0.7 and searched for in the Dictionary of Natural Products (Chapman & Hall/CRC) and the MOTO database (http://appliedbioinformatics.wur.nl/moto, accessed on 23 April 2021). MSn measurements were performed to obtain information on the fragment ions generated in the linear ion trap within the same analysis. Finally, metabolites were confirmed by comparing MS/MS spectra, with fragments reported in the literature as the main tool for the putative identification of OLC [20,24,29].

### 2.6. Statistical Analysis

Data are presented as the mean and standard deviation (SD). Statistical analysis was performed using SAS (version 9.4). The assumption of normalization was checked with standardized bias and standardized kurtosis. Statistical differences in the concentration of metabolites and OLC at different post-administration times (1, 2 and 4.5 h) for each compound and tissue were evaluated using one-way ANOVA, followed by the LSD post hoc test. Differences were considered significant at *p* < 0.05.

## 3. Results and Discussion

**Figure 1:**
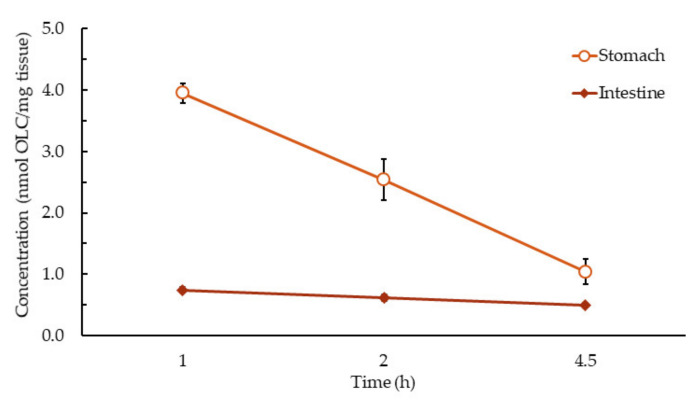
OLC concentrations in the small intestine and stomach (nmol/g tissue), calculated as nmol OLC equivalents. Results are expressed as the mean ± standard deviation.

OLC and its metabolites were not detected in the plasma and tissues of the control group. In the groups receiving ROO, OLC was only detected in the stomach and small intestine samples, with the maximum concentration (C_max_) being at 1 h of consumption (*p* < 0.05) (Figure 1). In the stomach samples, the concentration decreased by 36% and 74% at 2 h and 4.5 h, respectively, which could be due to gastric emptying and the fact that OLC decomposition increases with gastric residence time, although in the fasted (pH 1.5) and fed (pH 2–3) conditions and normal physiological time frames (up to 4 h), some would remain intact and enter the small intestine unhydrolyzed [39]. The concentration of the OLC that reached the intestine decreased by 16% and 33% at 2 h and 4.5 h post-ingestion, respectively (Figure 1), which can be attributed mainly to the intestinal metabolism and less to intestinal absorption [29].

### 3.1. Metabolite Identification

Ten metabolites arising from phase I and phase II reactions were identified in the rat plasma and tissues. Accurate and comprehensive identification by LC-ESI-LTQ-Orbitrap-MS showed that the OLC metabolites were widely distributed in different tissues. The corresponding molecular formulas, MS/MS fragments, mass measurement errors and retention times are shown in the Supporting Information (S1). In addition, an example of a chromatogram for each metabolite and the parent compound is shown in Figure S1.

#### 3.1.1. Phase I Metabolism

**Figure 2:**
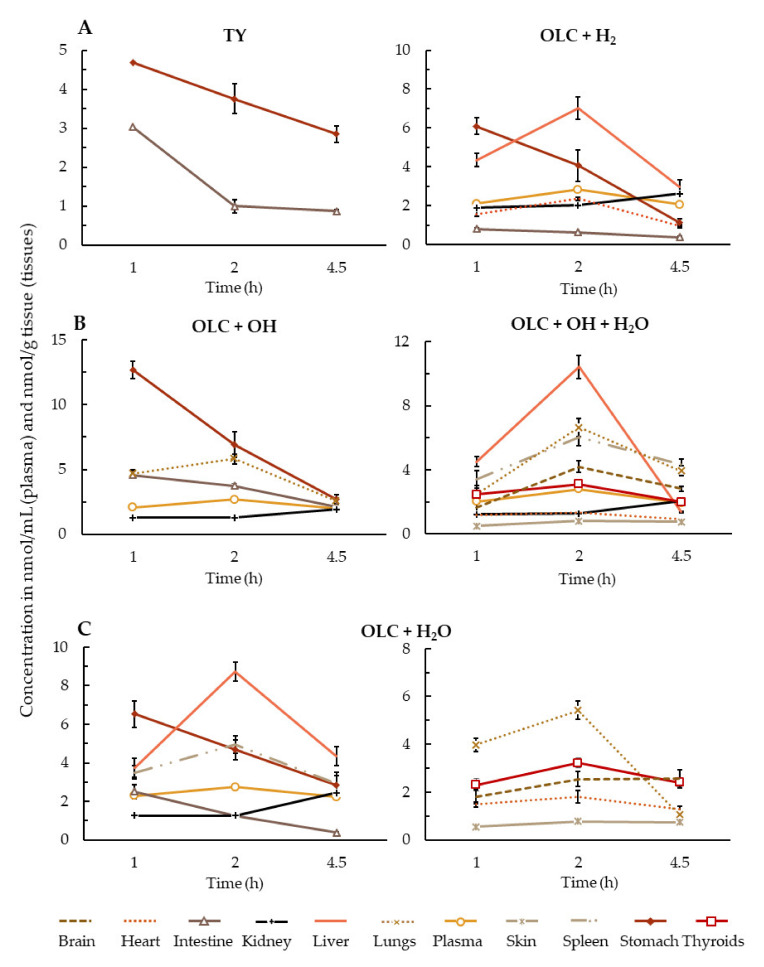
Phase I metabolites of OLC in plasma (nmol/mL) and tissues (nmol/g tissue), calculated as nmol OLC equivalents. (**A**): TY and OLC + H_2_ metabolites; (**B**): OLC + OH and OLC + OH + H_2_O metabolites; (**C**): OLC + H_2_O metabolite. Results are expressed as the mean ± standard deviation.

The first step in the digestion of dietary lipids in the stomach involves their dispersal into finely divided emulsion particles, generating a lipid–water interface [40]. Previous studies have demonstrated that phenolic compounds are transferred to the aqueous or oily phase according to their polarity [41]. In the case of OLC, its amphiphilic characteristics result in a partition between the oily and aqueous phase, tending toward a higher concentration in the latter (68.7%) due to its polar functional groups [42]. At the same time, OLC can be partially modified in the acidic environment of the stomach, where OLC was hydrolyzed into tyrosol (TY) and only detected in the stomach and intestinal samples, with the highest concentrations observed at 1 h of intragastric administration (Figure 2A). Reports on secoiridoid hydrolysis in the literature are contradictory. Thus, whereas in vitro and in vivo studies described secoiridoid compounds undergoing a rapid hydrolysis under gastric conditions, leading to an increase in hydroxytyrosol and TY concentrations [24,41], an in vitro digestion study observed good OLC stability in the gastric phase [42]. According to our results, the hydrolysis of OLC can occur due to either acidic conditions or the presence of nonlipolytic carboxylic ester hydrolases, which hydrolyze carboxylic esters cleaved into an acid and an alcohol [43]. In our study, the observation of TY in samples of the stomach and intestine but not the other organs can be attributed to its metabolization by enzymes of the gastrointestinal mucosal epithelium before entering the systemic circulation. However, TY metabolites were not detected, possibly because they were at a very low concentration.

Non-hydrolyzed OLC in the acidic conditions of the stomach can undergo different phase I biotransformations. The metabolite OLC + H_2_ was identified in the plasma, stomach, intestine, liver, kidney and heart samples (Figure 2A), with a C_max_ (the highest concentration observed among the three evaluation sample times) in the plasma, liver and heart at 2 h of ROO intake (*p* < 0.05), in the stomach and small intestine at 1 h and in the kidney at 4.5 h. In another study, small traces of OLC + H_2_ were detected in human urine at 2 h after a high intake of olive oil [20]. Recently, the same metabolite was identified in the plasma and lumen samples in an intestinal perfusion study in rats [29], whereas it was not found in a screening of secoiridoid metabolites in humans after an acute intake of EVOO [24]. Recently, it was suggested that the reduction OLC is catalyzed by NADPH-dependent aldo-keto reductases (AKR) located in the small intestine epithelium, and it can occur because OLC contains an open dialdehydic form of the attached elenolic acid molecule [29].

A hydroxylated metabolite, OLC + OH, was found in the plasma, stomach, intestine, kidney and lungs (Figure 2B). The C_max_ in the stomach and small intestine samples was at 1 h, at 2 h in the plasma and at 4.5 h in the lungs and in the kidney (*p* < 0.05). Only one previous study [29] has identified this metabolite in rat plasma and lumen samples, while in another it was reported in human urine, although there were doubts about whether it was in fact a hydroxytyrosol derivative such as oleacein [20]. In our study, OLC + OH was not detected in the main metabolizing organ, the liver, where it would have rapidly undergone other metabolic reactions, such as the addition of another OH group, methylation or glucuronidation. Its presence in the plasma, lungs and the brain could be due to enzymatic deconjugation, which occurs with glucuronidated metabolites of quercetin [44] or hydroxytyrosol [45].

The only metabolite present in all the samples was the hydrated form of OLC (Figure 2C). The C_max_ in the stomach and small intestine was at 1 h, in the kidney at 4.5 h (*p* < 0.05), in the brain and skin at 2 h and 4.5 h (the differences were insignificant) and in the plasma and the rest of the tissues at 2 h post-ingestion. Other studies have reported this metabolite in plasma [24,29].

A new OLC metabolite, OLC + OH + H_2_O, which was not previously reported in either human or animal experiments [20,23,24,25,29], was identified in all tissues except the stomach and small intestine (Figure 2B). The C_max_ in most of the samples was at 2 h post-consumption of ROO, at 4.5 h in the kidney and at both 2 h and 4.5 h in the skin. This metabolite could be formed from the hydroxylated and hydrated metabolites identified in the stomach and small intestine following further metabolic reactions in the liver (+ OH or + H_2_O). In our study, the presence of OLC + OH + H_2_O could be attributed to the hepatic expression of CYP enzymes responsible for the majority of phase I-dependent drug metabolism and for the metabolism of a huge variety of dietary constituents and endogenous chemicals [46]. 

In the stomach, small intestine and liver [47,48], CYP functions act as a barrier against drugs and chemical compounds, catalyzing and introducing an oxygen atom into substrate molecules, which often results in dealkylated and hydroxylated metabolites [48].

#### 3.1.2. Phase II Metabolism

**Figure 3:**
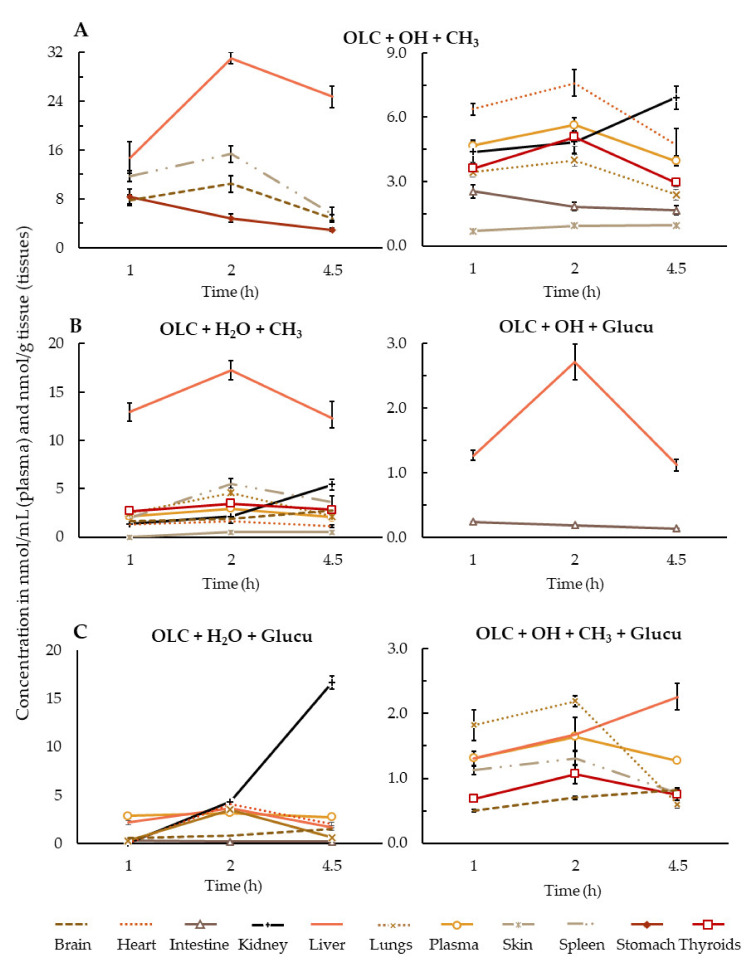
Phase II metabolites in plasma (nmol/mL) and tissues (nmol/g tissue), calculated as nmol OLC equivalents. (**A**): OLC + OH + CH_3_ metabolite; (**B**): OLC + H_2_O + CH_3_ and OLC + OH + glucuronidation metabolites; (**C**): OLC + H_2_O + glucuronidation and OLC + OH + CH_3_ + glucuronidation metabolites. Results are expressed as the mean ± standard deviation.

OLC that escapes first-pass metabolism and the phase I metabolites can undergo other biotransformations. Our results suggest that the hydroxylated (OLC + OH) and hydrated (OLC + H_2_O) forms of OLC were subject to the addition of a methyl group (OLC + OH + CH_3_ and OLC + H_2_O + CH_3_). Catechol-O-methyltransferases (COMT) are conjugating enzymes that catalyze the transfer of a methyl group from *S*-adenosyl-L-methionine to phenolic compounds with an O-diphenolic moiety. This reaction usually takes place at the 3´position of the compound, although a minor proportion of 4´-O-methylated product is also possible [49]. OLC + OH + CH_3_ was the only conjugated metabolite detected in all analyzed tissues (Figure 3A). The COMT enzymes are most active in the liver and kidneys but are also active in the pylorus of the stomach and in the small intestine, especially the duodenum and ileum [50], a behavior that would explain the presence of this conjugated metabolite in our samples. In contrast, OLC + H_2_O + CH_3_ was found in almost all samples, except in the stomach and intestine, probably due to the high activity of COMT enzymes in the liver. The C_max_ of both conjugated OLC derivatives (OLC + OH + CH_3_ and OLC + H_2_O + CH_3_) in the plasma and most of the tissues (liver, spleen, hearts, lungs and thyroids) were reached at 2 h after ROO ingestion (*p* < 0.05) and at 4.5 h in the kidney (*p* < 0.05). In the skin, the C_max_ was reached at 2 h and with a similar concentration at 4.5 h (*p* > 0.05). The only previous study to determine the intestinal metabolic profile of OLC did not detect either of these metabolites [29], whereas other in vivo studies observed the same trend found here [20,23,24,25].

A frequent phase II reaction is catalyzed by uridine-disphosphate glucuronosyl transferase (UGT), an enzyme located in the endoplasmic reticulum that mediates the transfer of glucuronic acid from uridine diphosphate to lipophilic compounds [51]. It is known that UGT interacts with structures that have one glucuronidation site and two hydrophobic sites separated by 3 or 6.2 Å of aliphatic hydroxyls, carboxylic acid and amines [52,53]. Although the liver is the main site of glucuronidation [54], there is clear evidence for the expression of UGT in the stomach [54], and UGT1A8 and UGT1A10 are exclusively expressed in the small intestine [53]. Figure 3 also shows the metabolites containing a glucuronic acid. OLC + OH + glucu was only detected in the small intestine, where UGT enzymes are expressed, and the liver (Figure 3B). The C_max_ in the small intestine was at 1 h after ROO intake, and in the liver, it was at 2 h (*p* < 0.05). In contrast, OLC + H_2_O + glucu was more widespread (Figure 3C), with a C_max_ in the small intestine at 1 h of ROO intake, at 2 h in the plasma, liver, heart and lungs and at 4.5 h in the kidney and brain (*p* < 0.05). OLC + H_2_O + CH_3_ or OLC + H_2_O + glucu underwent further metabolization, resulting in OLC + OH + CH_3_ + glucu, which was found in the plasma, liver, spleen, lungs, thyroid and brain (Figure 3C). The three phase II metabolites detected in our samples had not been previously identified in rat tissues [25] or human urine [20,22,24] or plasma [23]. In a recent study on OLC absorption, only the OLC + H_2_O + glucu metabolite was identified in plasma [29]. Nevertheless, the glucuronidation of OLC, which was not observed here, had been detected in human urine after the intake of EVOO [20,24].

### 3.2. Brief Summary of OLC Metabolite Distribution by Tissue

Our results show that the OLC metabolites were widely distributed in all the tested tissues, even in the brain and skin (Figure 2 and Figure 3). The liver, small intestine and plasma were the biological samples with the greatest variety of metabolites.

In the stomach samples, in addition to OLC, four metabolites from the phase I reactions and one from the phase II reactions were detected (Figure 2 and Figure 3). The presence of a TY metabolite in the stomach indicates that OLC underwent a rapid hydrolysis under gastric conditions. The C_max_ of all the metabolites was observed at 1 h of ROO intake.

Besides OLC, a wide range of its metabolites was found in the small intestine, where concentrations were lower than in the stomach. It is well known that the small intestine is the main site where xenobiotic absorption occurs [55,56], and the intestinal epithelial membrane is the principle physiological barrier that chemicals must cross to enter the bloodstream [57]. Phenolic compounds can be metabolized in the small intestine by numerous pathways involving both phase I and phase II reactions. Accordingly, four phase I and three phase II derivatives were identified (Figure 2 and Figure 3). For all the metabolites, the C_max_ was observed at 1 h of ROO intake. These results confirm that OLC is subjected to a high intestinal metabolism, as previously reported [29].

According to the results obtained, the liver (in addition to the small intestine) represents the main metabolizing organ for OLC. For most of the metabolites detected in the liver, the C_max_ was at 2 h. Up to five phase II metabolites were found at high concentrations, in accordance with the functionality of this organ in xenobiotic and phenolic compound metabolism. The results are consistent with those obtained in a tissue distribution study in rats after olive cake ingestion, in which the highest number and concentration of metabolites were detected in the liver [25].

OLC was not found in the plasma, which could be explained by its relatively low absorption (16%) and high intestinal metabolism [29]. In the latter study, OLC was detected in mesenteric blood plasma, but the compound was administered directly to the small intestine, thus avoiding passing through the stomach and the liver, where OLC hydrolysis and metabolism can potentially take place, respectively. Although OLC was not detected in the plasma, four phase I and four phase II metabolites were identified in the plasma with a C_max_ at 2 h. This study demonstrates for the first time that OLC + OH + CH_3_ is the main circulating OLC metabolite in rats after OLC consumption, followed by OLC + H_2_O + glucu. The systemic exposure of these metabolites can reflect tissue exposure [58]. It is worth noting that most in vitro and in vivo studies or clinical trials determining the effects of OLC have focused on the control and progression of different types of cancer, cardiovascular diseases, neurodegeneration, anti-aging processes and immunoinflammatory diseases, but the biological concentrations of OLC [6,59] and its major circulating metabolites were not considered.

The potential protective effect of OLC againt Alzheimerʹs disease has been investigated in both in vitro and in vivo studies [13,14,60]. However, for the first time, it was demonstrated that only the metabolites (Figure 2 and Figure 3) were able to cross the blood–brain barrier in rats—at the dose of OLC administered—and undergo brain uptake. The results showed brain accumulation of the OLC + OH + CH_3_ metabolite, suggesting that this metabolite could be able to exert beneficial effects, specifically neuroprotective activity, by the reduction of the oxidative stress at the neuronal level. However, in the last decade, several studies have been focused on the evaluation of the potential health benefits of OLC, and the data available about the biological relevance of its metabolites are still limited. Futures studies about the effective brain accumulation of the main biological OLC metabolites are needed to determine their potential to protect neuronal cells.

Based on the number and high concentration of OLC metabolites detected in the lungs, this organ is an important site of extrahepatic OLC metabolism. It is well known that the lungs contain drug-metabolizing enzymes, with low levels of monooxidases, transferases and esterases found in the endothelial cells, which may contribute to the elimination of drugs and xenobiotics [60,61]. A wide range of OLC derivatives (six) were also found in the heart, more than in the spleen and thyroids (5). In all these samples, OLC + OH + CH_3_ was the main metabolite detected. Various drug-metabolizing enzymes are also expressed by macrophages and lymphocytes present in the white pulp region of the spleen, explaining the presence of metabolites in this tissue. More information about the metabolite functionality in the spleen is required [25], although a role of the spleen in lipid metabolism has been reported [62].

The wide range of metabolites detected in the kidney indicates that renal excretion is a major pathway of elimination for OLC metabolites in rats. In addition, accumulating evidence indicates that specific CYP, UGT and possibly other drug-metabolizing enzymes contribute to renal drug and chemical metabolic clearance, thereby modulating intrarenal exposure to these compounds, as well as regulating the activity of physiological mediators [63].

Even though the skin was the tissue where the fewest metabolites arrived, this study also contributes to providing a new perspective for understanding how the mechanism through EVOO phenolic compounds acts in the body.

The findings found in this study suppose a breakthrough in the research to understand the positive correlation of olive oil phenolic compound consumption and the prevention and improvement of some diseases. Nevertheless, further experiments are required to investigate the effect of the main circulating metabolites on the functions of the target tissues, such as those in the brain, heart, skin and spleen.

## 4. Conclusions and Remarks

There is still a lack of information on the distribution and accumulation of olive oil phenolic compounds in the body. In this study, the distribution and accumulation of OLC metabolites in rat tissues was studied for the first time. The results demonstrate that under gastric conditions, OLC was hydrolyzed to TY, whereas the non-hydrolyzed OLC underwent different phase I biotransformations in the stomach and small intestine. Once absorbed, the phase I metabolites were widely distributed throughout the organism or underwent further metabolic reactions. Although OLC was not detected in the tissues, its metabolites were found in the lungs, heart, brain, thyroids, spleen and skin in sufficient concentrations to exert beneficial effects. The main circulating metabolites arising from phase II reactions were OLC + OH + CH_3_ and OLC + H_2_O + glucu, suggesting that these metabolites are likely to significantly contribute to the beneficial health effects associated with the regular consumption of olive products. However, more studies are necessary to determine the concentrations and composition of OLC metabolites in human plasma and tissues, as well as pharmacodynamic studies that relate concentrations vs. effects.

## Data Availability

Data is contained within the article or supplementary material. The data presented in this study are available in Figure 1, Figure 2 and Figure 3.

## Supplementary Materials

The following are available online at https://www.mdpi.com/article/10.3390/antiox10050688/s1, Table S1. Identification of OLC and its metabolites in plasma and tissues through LTQ-Orbitrap-MS. Figure S1. Extracted ion chromatograms of the OLC and its identified metabolites in different tissues.
