# Supplementary material for: Tissue Distribution of Oleocanthal and Its Metabolites after Oral Ingestion in Rats
Source: Antioxidants (Basel). 2021 Apr 27;10(5):688. doi: 10.3390/antiox10050688 (PMC8146289; doi:10.3390/antiox10050688)
Supplement: Supplementary file 1 [file antioxidants-10-00688-s001.zip › antioxidants-1196440-supplementary.pdf]

Table S1. Identification of OLC and its metabolites in plasma and tissues through LTQ-Orbitrap-MS.

Metabolite description	Elemental composition [M - H]	RT (min)	Observed [M - H]	Exact mass	Mass error (ppm)	Major fragments
OLC	$C_{17}H_{19}O_5$	7.26	303.1237	303.1227	1	285/179
Tyrosol	$C_8H_9O_2$	4.55	137.0606	137.0597	0.894	93
OLC + H_2	$C_{17}H_{21}O_5$	7.97	305.1403	305.1383	0.05	174/205
OLC + OH	$C_{17}H_{19}O_6$	6.81	319.1184	319.1176	0.785	153/183
OLC + OH + H_2O	$C_{17}H_{21}O_7$	7.05	337.1314	337.1281	3.221	295/201
OLC + H_2O	$C_{17}H_{21}O_6$	6.76	321.1337	321.1332	0.435	201/183
OLC + OH + CH_3	$C_{18}H_{21}O_6$	8.96	333.1347	333.1332	0.835	165/183
OLC + H_2O + CH_3	$C_{18}H_{23}O_6$	7.31	335.1492	335.1489	0.285	151/201
OLC + OH + Glucu	$C_{23}H_{27}O_{12}$	9.62	495.1502	495.1497	0.458	197
OLC + H_2O + Glucu	$C_{23}H_{29}O_{12}$	6.47	497.1663	497.1653	0.947	321/201
OLC + H_2O + CH_3 + Glucu	$C_{24}H_{31}O_{13}$	6.57	527.1801	527.1759	4.1974	351/183

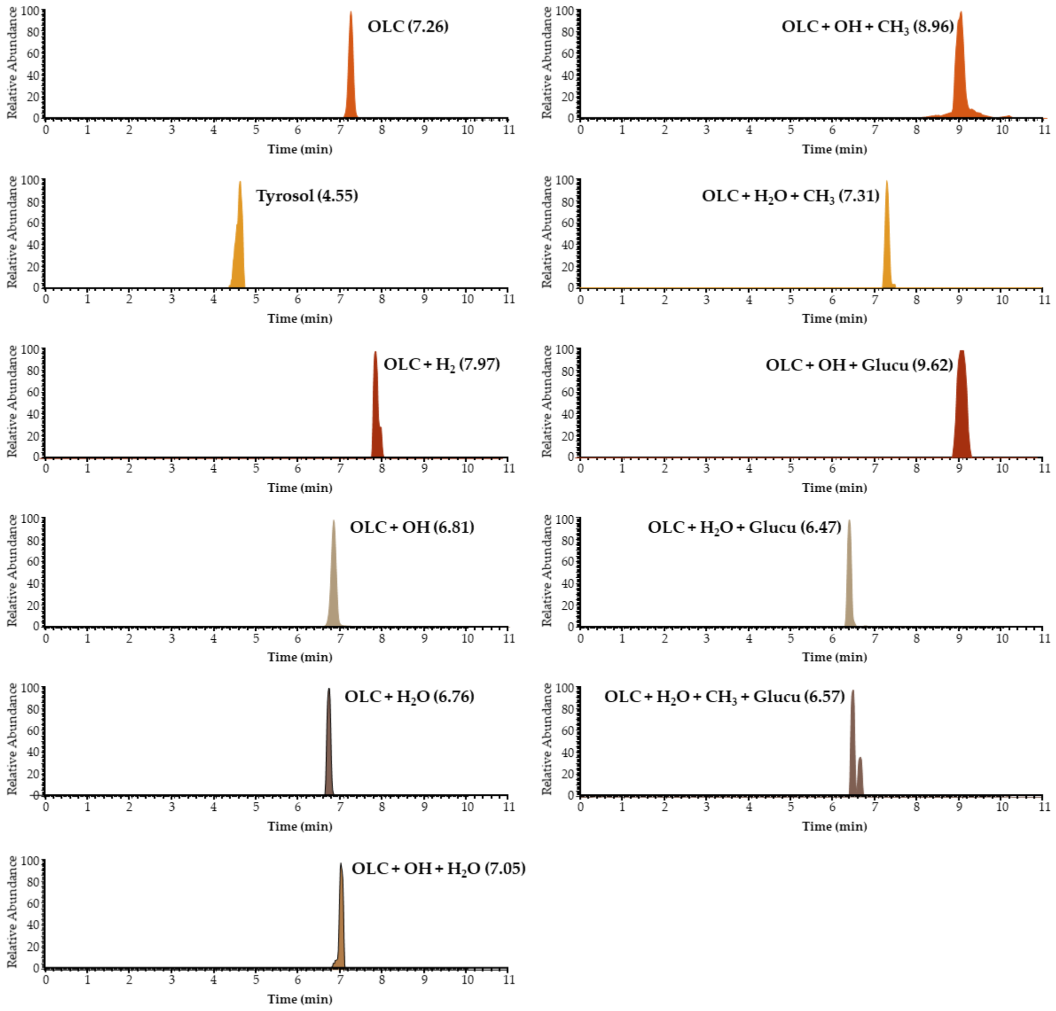

Figure S1. Extracted ion chromatograms of the OLC and its identified metabolites in different tissues.
